# Regulation of Microglia and Macrophage Polarization via Apoptosis Signal-Regulating Kinase 1 Silencing after Ischemic/Hypoxic Injury

**DOI:** 10.3389/fnmol.2017.00261

**Published:** 2017-08-14

**Authors:** So Yeong Cheon, Eun Jung Kim, Jeong Min Kim, Eun Hee Kam, Byung Woong Ko, Bon-Nyeo Koo

**Affiliations:** ^1^Department of Anesthesiology and Pain Medicine, Yonsei University College of Medicine Seoul, South Korea; ^2^Anesthesia and Pain Research Institute, Yonsei University College of Medicine Seoul, South Korea

**Keywords:** apoptosis signal-regulating kinase 1 (ASK1), M1/M2 polarization, BV2 microglia cell line, RAW264.7 macrophage cell line, cerebral ischemia, hypoxia, late inflammation, ischemic stroke

## Abstract

Inflammation is implicated in ischemic stroke and is involved in abnormal homeostasis. Activation of the immune system leads to breakdown of the blood–brain barrier and, thereby, infiltration of immune cells into the brain. Upon cerebral ischemia, infiltrated macrophages and microglia (resident CNS immune cell) are activated, change their phenotype to M1 or M2 based on the microenvironment, migrate toward damaged tissue, and are involved in repair or damage. Those of M1 phenotype release pro-inflammatory mediators, which are associated with tissue damage, while those of M2 phenotype release anti-inflammatory mediators, which are related to tissue recovery. Moreover, late inflammation continually stimulates immune cell infiltration and leads to brain infarction. Therefore, regulation of M1/M2 phenotypes under persistent inflammatory conditions after cerebral ischemia is important for brain repair. Herein, we focus on apoptosis signal-regulating kinase 1 (ASK1), which is involved in apoptotic cell death, brain infarction, and production of inflammatory mediators after cerebral ischemia. We hypothesized that ASK1 is involved in the polarization of M1/M2 phenotype and the function of microglia and macrophage during the late stage of ischemia/hypoxia. We investigated the effects of ASK1 in mice subjected to middle cerebral artery occlusion and on BV2 microglia and RAW264.7 macrophage cell lines subjected to oxygen-glucose deprivation. Our results showed that ASK1 silencing effectively reduced Iba-1 or CD11b-positive cells in ischemic areas, suppressed pro-inflammatory cytokines, and increased anti-inflammatory mediator levels at 7 days after cerebral ischemia. In cultured microglia and macrophages, ASK1 inhibition, induced by NQDI-1 drug, decreased the expression and release of M1-associated factors and increased those of M2-associated factors after hypoxia/reperfusion (H/R). At the gene level, ASK1 inhibition suppressed M1-associated genes and augmented M2-associated genes. In gap closure assay, ASK1 inhibition reduced the migration rate of microglia and macrophages after H/R. Taken together, our results provide new information that suggests ASK1 controls the polarization of M1/M2 and the function of microglia and macrophage under sustained-inflammatory conditions. Regulation of persistent inflammation via M1/M2 polarization by ASK1 is a novel strategy for repair after ischemic stroke.

## Introduction

Inflammation is a key contributor to the pathophysiology of ischemic stroke and is responsible for abnormal homeostasis (Amantea et al., 2009; Denes et al., 2010; Iadecola and Anrather, 2011). After ischemic insults, activation of the immune system causes leakiness of the blood–brain barrier (BBB), and immune cells such as macrophages, neutrophils and leukocytes, infiltrate the lesion area via the disrupted BBB (Amantea et al., 2009; Denes et al., 2010; Iadecola and Anrather, 2011). Recruitment of immune cells results in initiation of inflammatory processes in the brain parenchyma, which include both tissue damage and repair (Fu et al., 2015). Although inflammation is involved in all phases of ischemic cascade (Iadecola and Anrather, 2011), late inflammation in the brain, particularly after ischemic stroke, provokes persistent immune cell recruitment and eventually aggravates cerebral infarcts (Mena et al., 2004; Vogelgesang et al., 2014). Therefore, regulation of late inflammation in ischemic stroke is considered important to prevent development of subsequent brain damage.

Once activated, microglia and infiltrated macrophages are able to modify their morphology and function by switching between M1 and M2 phenotypes (Tugal et al., 2013; Sica et al., 2015). A classical (M1) polarized phenotype secretes Th1 cytokines and pro-inflammatory mediators, such as interleukin (IL)-6, IL-1β, and tumor necrosis factor (TNF)-α, which are involved in tissue damage and prolong the neuro-inflammatory response (Tugal et al., 2013; Sica et al., 2015; Qin et al., 2016). The alternative (M2) phenotype releases Th2 cytokines and anti-inflammatory mediators including transforming growth factor (TGF)-β, IL-4, IL-10, and arginase (arg-1), which are associated with prevention of inflammation, tissue repair, and neuro-protection (Tugal et al., 2013; Sica et al., 2015; Qin et al., 2016). Although little is known about the relationship between the chronic phase of cerebral ischemia and immune cell phenotype modulating the exaggerated inflammatory environment after ischemic stroke via regulation of M1/M2 polarization could be a therapeutic aim.

After I/R injury, the excessive accumulation of reactive oxygen species (ROS) in cells induces oxidative stress and subsequent damage (Cheon et al., 2013). ROS is a key player in the pathophysiology of cerebral ischemia and ASK1 is known as the initial responder of ROS (Kim et al., 2011; Cheon et al., 2016b). Also, ASK1 is activated by various stimuli, such as oxidative stress, calcium overload, and receptor-mediated inflammatory signals (Hayakawa et al., 2006; Takeda et al., 2008). After ischemic insult, ASK1 immediately promotes intracellular signaling and induces apoptotic cell death, and down-regulation of ASK1 diminishes cell death and cerebral infarct. Previous studies demonstrated that ASK1 is associated with brain edema (Kim et al., 2011; Song et al., 2015). In addition, in mammals, ASK1 has been shown to modify innate immunity and to be required for inflammatory response (Matsukawa et al., 2004; Hayakawa et al., 2006; Takeda et al., 2008). ASK1 deficiency leads to suppression of lipopolysaccharide (LPS)-induced inflammatory cytokine production, such as IL-6, IL-1β, and TNF-α in splenocytes and bone marrow-derived dendritic cells (BMDCs). Also, in an LPS-induced sepsis model, ASK1-deficient mice shows resistance against LPS injury (Matsukawa et al., 2004; Takeda et al., 2008). Further, in primary microglia cell culture, ASK1 is found to be closely related to TNF-α and iNOS production (Katome et al., 2013). Class A scavenger receptor suppresses M1 macrophages, thereby reducing IL-6, IL-1β, and TNF-α in accordance with the dampening of ASK1/p38/NF-κB pathway (Hu et al., 2011). In this study, we focused on ASK1 and we hypothesized that ASK1 is likely to affect inflammation via modulation of M1/M2 polarization after I/R injury. Therefore, we examined whether ASK1 could modulate polarization of microglia and macrophage in mice after ischemic injury and in cultured BV2 microglia and RAW264.7 macrophage cell lines after hypoxic injury.

## Materials and Methods

### Mouse Focal Cerebral Ischemia Model

All animal experiments in this study were conducted in accordance by the Guide for the Care and Use of Laboratory Animal Care and were approved by Institutional Animal Care and Use Committee (IACUC) in Yonsei University. Mice were provided free access to chow and water under a 12-h dark and light cycle. A total of 52, adult male C57BL/6 mice aged 8–12 weeks (Orient, Seongnam, Korea) were used in this study. To mimic ischemic stroke, we utilized a middle cerebral occlusion (MCAO) model. Anesthesia was induced with 5% isoflurane and maintained with 2% isoflurane in mixed gas. Mice were placed onto a homeothermic blanket, and a longitudinal incision was made along the midline of the neck. MCA was occluded with a 6-0 blue nylon suture for 1 h and mice were reperfused for 7 days. At 7 days after MCAO, Zoletil mixture (30 mg/kg; active ingredient: Zolezepam and Tiletamin; Virbac Laboratories, Carros, France) was injected intraperitoneally. Mice were cardiac-perfused with saline, and brains were isolated for immunohistochemistry and ELISA assay. The mice were divided into three groups: normal group (control), ischemia/reperfusion (I/R) group, and ischemia/reperfusion + si-ASK1 treated (I/R+si-ASK1) group.

### siRNA Experiment

ASK1-siRNA was purchased from Ambion [Austin, TX, United States; sense, 5′-GCUGGUAAUUUAUACACuGtt-3′; antisense, 5′-CAGUGUAUAAAUUACGAGCtt–3′; conc, 5 μM (178.5–192.3 nM/g)] (Cheon et al., 2016b; Cho et al., 2016). Based on a previous study, a 100-μl solution mixed with siPORT*NeoFX* (Ambion) and ASK1-siRNA was administrated into the left ventricle of the mice by an osmotic pump with a brain infusion kit (Alzet, Cupertino, CA, United States) for 3 days before MCAO. The osmotic pump was planted subcutaneously on the dorsal side, and brain infusion cannula connected to the osmotic pump was placed on the left ventricle (mediolateral 1.0 mm, anteroposterior 0.2 mm, dorsoventral 3.0 mm). The hole in the skull was made by using a drill (Cheon et al., 2016b; Cho et al., 2016).

### BV2 Microglia and RAW 264.7 Macrophage Cell Cultures

Murine brain microglia (BV2 cell line) were cultured with RPMI 1640 (Hyclone^TM^, GE Healthcare Life Sciences, Logan, UT, United States) containing 10% fetal bovine serum (FBS, GE Healthcare Life Sciences) and 1% penicillin-streptomycin solution (Thermo Scientific, Waltham, MA, United States). Murine macrophages (RAW 264.7cell line) were cultured with DMEM high glucose cultured media (Hyclone^TM^, GE Healthcare Life Sciences) containing 10% FBS (GE Healthcare Life Sciences) and 1% penicillin-streptomycin solution (Thermo Scientific) at 37°C. The cultured cells were incubated in a humid atmosphere under the presence of 5% CO_2_ at 37°C.

### Oxygen/Glucose Deprivation

To induce oxygen and glucose deprivation, microglia and macrophage cultures were transferred to an anaerobic chamber after being washed with phosphate buffer saline (PBS). Transferred cells were then cultured in deoxygenated glucose-free balanced solution (BSS_0_) containing 5.36 mM KCl, 0.81 mM NaH_2_PO_4_, 0.81 mM MgSO_4_, and116 mM NaCl, and incubated for 4 h in a 37°C anaerobic chamber. After 4 h, the cells were washed with PBS, the cultured media was changed, and cells were incubated for 24 h under the presence of 5% CO_2_ at 37°C. To inhibit ASK1, the ASK1 inhibitor NQDI-1 drug (600 nM, Tocris Bioscience, Bristol, United Kingdom) was used in this study and was applied 1 h before hypoxia and 4 h during hypoxia.

### Immunofluorescence Staining

Mice were cardiac-perfused with 4% formaldehyde, and brains specimens were fixed with 4% formaldehyde for 24 h. Fixed brains were immersed in 30% sucrose for 2 days and then frozen with OCT compound (Sakura Finetek Japan Co., Ltd., Tokyo, Japan) at -70°C in a deep freezer. Frozen brain tissues were sectioned coronally with a cryotome with 20-μm thickness and onto coated slide glass. After drying the slide at room temperature, sections were treated with Trion-X 100 (0.3%) for permeability over 1 h and treated blocking solution [5% bovine serum albumin (BSA)] at room temperature for 1 h. After washing with PBS, primary antibodies such as anti-Iba-1 (Abcam, Cambridge, United Kingdom), anti-CD11b (Millipore, Bedford, MA, United States), anti-CD206 (Abcam), and anti-ASK1 (Santa Cruz Biotechnology, Santa Cruz, CA, United States) were incubated, respectively, overnight at 4°C. Secondary antibody conjugated FITC or Rhodamine (Jackson ImmunoResearch Laboratories, West Grove, PA, United States) was used and incubated for 1 h at room temperature after washing with PBS. Slides were mounted with Vectashield with DAPI (Vector Laboratories Inc., Burlingame, CA, United States). The specimens were observed by using an LSM 700 confocal microscope (Carl Zeiss, Thornwood, NY, United States) and microscope (Olympus, Tokyo, Japan). Immunoreactivities for CD11b, Iba-1, CD206, and ASK1 were measured using ImageJ (ImageJ, Bethesda, MD, United States) and Zen 2010 software (Carl Zeiss) by analyzing the mean intensities, which apply a manual threshold above the background intensity. The mean intensities were analyzed by average intensity, obtained from three slice of each mouse. Three fields of the cortex, striatum, and hippocampus were randomly chosen from each specimen.

### Cresyl Violet Staining

Brains of mice were isolated and fixed with 4% formaldehyde and sectioned coronally with a cryotome at a thickness of 20-μm. Sectioned brains were stained with filtered cresyl violet acetate (0.5%) (Sigma–Aldrich, St. Louis, MO, United States), which was dissolved in 300 mL distilled water with 10% glacial acetic acid. Slides were incubated with cresyl violet solution for 3 min at room temperature and coverslipped with permanent mounting medium (Vector Laboratories), and then, samples were observed under a microscope (Olympus).

### Enzyme-Linked Immunosorbent Assay (ELISA)

To evaluated cytokine levels from tissue, BV2 microglia cell line, BV2 microglial supernatants, RAW 264.7 macrophage cell line and RAW264.7 macrophage supernatants, ELISA assays were performed according to commercial protocols. Levels of pro-inflammatory cytokines, such as IL-1β, IL-6, and TNF-α, and anti-inflammatory cytokines including IL-10, were measured. Samples were analyzed by High Sensitivity mouse IL-1 beta/IL-1F2, IL-6, TNF-alpha, and IL-10 Quantikine ELISA kits (R&D systems, Minneapolis, MN, United States). Following the manufacturer’s instructions, reagents and standards in the ELISA kits were prepared, and prepared sample was added to each well with assay diluents and incubated. After washing with wash buffer for three times, samples were incubated with mouse IL-1β, IL-6, TNF-α, or IL-10 conjugates for 2 h at room temperature. After reactions completed, samples were read at a 450-nm wavelength by an ELISA reader.

### Real-Time-PCR

To evaluate M1 and M2 phenotypes, BV2 microglia and RAW 264.7 macrophage cell lines were collected from which total RNA was extracted by using RNeasy^®^ Mini Kit (QIAGEN, Austin, TX, United States). RNA concentration was assessed using NanoDrop^®^ ND-1000 (Thermo Scientific, NanoDrop Technologies, Wilmington, DE, United States). Real-time PCR was performed using the one-step SYBR PrimeScript RT-PCR Kit II (Perfect Real Time) (Takara Bio Inc., JAPAN) on an ABI StepOne Plus. PCR was performed in a total reaction mixture volume of 20 μl, composed of One step SYBR RT-PCR Buffer, PrimeScript 1 step Enzyme Mix 2, ROX Reference Dye, each forward primer (100 nM), each reverse primer (100 nM), and sample RNA diluted in RNase Free dH_2_O. The RNA samples were used by 10-fold serial dilution. The primers were as follows: TNF-α: Forward (F) 5′-ACGGCATGGATCTCAAAGAC-3′, Reverse (R) 5′-AGATAGCAAATCGGCTGACG-3′, IL-1β: (F) 5′-TGTCTTGGCCGAGGACTAAGG-3′, (R) 5′-TGGGCTGGACTGTTTCTAATGC-3′, IL-6: (F) 5′-TCCAGTTGCCTTCTTGGGAC-3′, (R) 5′-GTGTAATTAAGCCTCCGACTTG-3′, iNOS: (F) 5′- CAGCTGGGCTGTACAAACCTT-3′, (R) 5′-CATTGGAAGTGAAGCGTTTCG-3′, Cxcl10: (F) 5′-GGATGGCTGTCCTAGCTCTG-3′, (R) 5′-TGAGCTAGGGAGGACAAGGA-3′, IL-10: (F) 5′-GCTCTTACTGACTGGCATGAG-3′, (R) 5′-CGCAGCTCTAGGAGCATGTG-3′, Ym-1: (F) 5′-GGGCATACCTTTATCCTGAG-3′, (R) 5′-CCACTGAAGTCATCCATGTC-3′, ASK1: (F) 5′-AGGACGGAGACTGTGAGGGT-3′, (R) 5′-GTCCTGCATAGACGATCCCAT-3′, GAPDH: (F) 5′-CCATTTGCAGTGGCAAAG-3′, (R) 5′-CACCCCATTTGATGTTAGTG-3′. The standard curve and melt curve analysis were used and cycling threshold (*C*t) values were normalized to *C*t values of housekeeping gene. The values were presented by relative quantity (RQ). The results were analyzed by StepOnesoftware v2.3.

### Gap Closure Assay

Microglia or macrophages were seeded in 60-mm dishes 1 day prior to experimentation. A gap was made with a sterile, 1000-μl, blue pipette tip, and dishes were washed with DPBS to remove cell culture media and cell debris. Cultured cells underwent oxygen and glucose deprivation for 4 h and were transferred to normal culture media in a 37°C anaerobic chamber. Cells were incubated for 24 h in a 37°C anaerobic chamber, and images were obtained using inverted microscope with a digital camera (Leica, MC179, Wetzlar, Germany).

### Flow Cytometry Analysis of BV2 Microglia and RAW 264.7 Macrophage Cell Lines

BV2 microglia and RAW264.7 macrophage cell line were collected and washed with DPBS. FACS buffer [Dulbecco’s Phosphate-Buffered Saline (PBS) with 2% Fetal Bovine Serum, 0.09% Sodium Azide] (BD Pharmingen^TM^, BD Biosciences, San Hose, CA, United States) were added to cells and incubated for 10 min. Next, cells were centrifuged at 250 *g* for 3 min, and the pellet was resuspended with 500 μl of FACS buffer. Cells were blocked with mouse Fc blocking solution (1:50, BD Biosciences) for 10 min on ice and centrifuged at 250 *g* for 3 min at 4°C. Cells were then stained with the following antibodies: mouse anti-CD40-FITC (1:50 MACS Miltenyi Biotec, Bergisch Gladbach, Germany) and mouse anti-CD206-PE (1:50, BD Pharmingen^TM^, BD biosciences) for 30 min on ice. Cell suspensions were then filtered through a cell strainer with a 40-μm nylon mesh. Cell fluorescence was acquired using flow cytometry with a LSR II analyzer (BD Pharmingen^TM^, BD biosciences). Data were analyzed using FlowJo software, version10 (FLOWJO, LLC, Ashland, OR, United States).

### TUNEL Assay

Terminal deoxynucleotidyl transferase dUTP nick end labeling (TUNEL) assay was performed to evaluate DNA fragmentation. We used TUNEL assay kit, purchased from Roche Diagnostics (Indianapolis, IN, United States), and performed according to the manufacturer’s manual. Counterstaining was performed with propidium iodide (PI) (Sigma–Aldrich) and the number of TUNEL-positive cells was counted in three 100 μm × 100 μm squares of each specimen and expressed as the number of TUNEL-positive cells square millimeter.

### Statistical Analysis

Data are presented as the mean ± standard deviation (SD). Statistical comparisons between the groups were assessed with non-parametric Mann–Whitney test (Prism version 5.0 software, GraphPad Software, San Diego, CA, United States). Statistical significance among groups was assigned to *p*-values less than 0.05.

## Results

### Increase in Microglia/Infiltrated Macrophages Reduced in the Ischemic Cortex and Striatum after ASK1 Silencing

**FIGURE 1:**
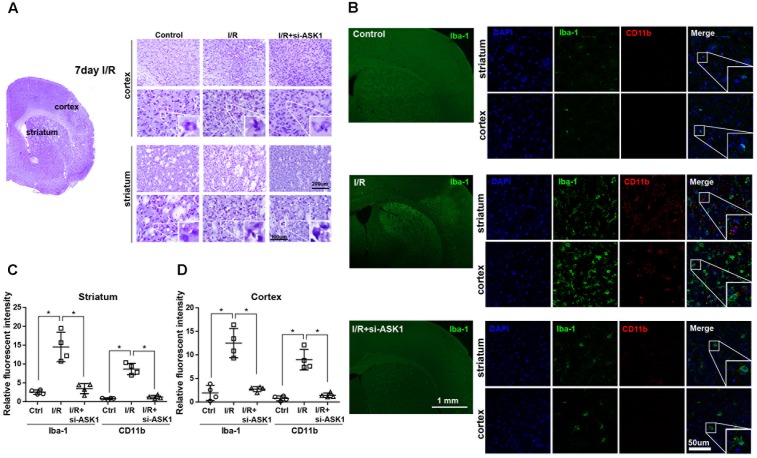
Decreased microglia and macrophage markers in the striatum and cortex, respectively, upon ASK1 silencing at 7 days after cerebral ischemia. **(A)** Cresyl violet staining for cell histology in the cortex and striatum shows that I/R-induced cell loss was not detected at 7 days in the I/R+si-ASK1 group. **(B)** Immunofluorescent staining for Iba-1 (green) and CD11b (red) in the cortex and striatum reveals that I/R-induced increase in Iba-1 or CD11b-positive cells were reduced in the I/R+si-ASK1 group at 7 days. **(C)** The graph shows the relative fluorescent intensities for Iba-1 and CD11b in the striatum (*n* = 4). **(D)** The graph indicates the relative fluorescent intensities for Iba-1 and CD11b in the cortex (*n* = 4). Values are means ± SD. Statistical comparisons between the groups were assessed with non-parametric Mann–Whitney test (^∗^*p* < 0.05, ^∗∗^*p* < 0.01, ^∗∗∗^*p* < 0.001 vs. I/R group). Statistical parameter (Supplementary Table 1). I/R, ischemia/reperfusion.

The previous study showed that siRNA for ASK1 significantly reduces ASK1 level (Kim et al., 2011; Cheon et al., 2016b). In this study, we followed this method and confirmed immunohistochemistry for ASK1 with/without administration of siRNA for ASK1 at 7 days after I/R. We observed that ischemia-induced ASK1 expression was efficiently reduced after ASK1 silencing (Supplementary Figure 1A). In addition, at 3 days after I/R, we performed real-time PCR for ASK1. Upregulated ASK1 transcripts after I/R significantly diminished after ASK1 silencing (Supplementary Figure 1B). Ischemic injury induces a functional change in the brain and leads to cell death, the morphological changes of which were observed. To observe cell morphology after cerebral ischemia, we performed cresyl violet staining 7 days after (I/R) (**Figure 1A**). As shown in **Figure 1A**, cell bodies observed in the cortex and striatum in the I/R group were smaller and thinner than those in the control group. After silencing ASK1, we observed comparatively rounder and healthier cell bodies, compared with those of the I/R group, at 7 days after I/R. To examine microglia and infiltrated macrophages, we used microglia and macrophage markers, such as Iba-1 and CD11b, respectively (**Figure 1B**). In the control group, Iba-1-positive and CD11b-positive cells were rarely observed in the cortex and striatum. However, Iba-1-positive and CD11b-positive cells were densely expressed in the I/R group. After silencing ASK1, Iba-1-positive and CD11b-positive cells decreased in the cortex and striatum, respectively, despite ischemic injury. The graphs for relative fluorescent intensity showed increased intensities for Iba-1 and CD11b were efficiently decreased after silencing ASK1, compared with the I/R group (**Figures 1C,D**). Therefore, our data showed that ASK1 silencing results in reduced microglia and macrophages in the ischemic cortex and striatum, respectively. Statistical parameters were shown in Supplementary Table 1.

### Enhanced Microglia/Infiltrated Macrophages Eliminated in Ischemic Hippocampus Tissue after ASK1 Silencing

**FIGURE 2:**
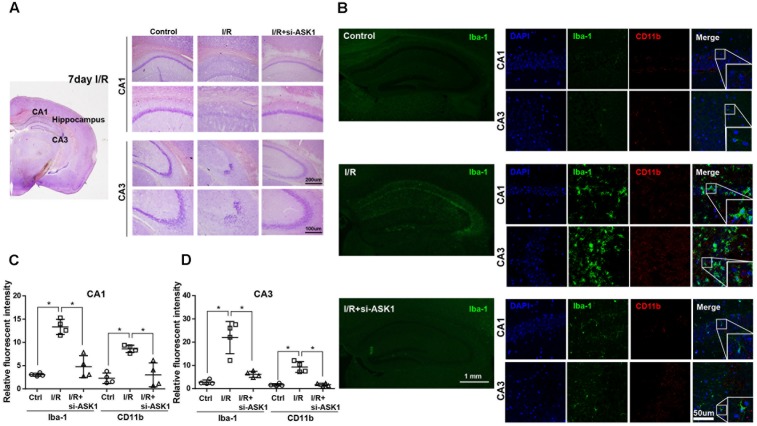
Reduced microglia and macrophage markers in the hippocampus upon silence of ASK1 at 7 days after cerebral ischemia. **(A)** Cresyl violet staining for cell histology in the hippocampus shows that I/R-induced cell loss was not detected at 7 days in the I/R+si-ASK1 group. **(B)** Immunofluorescent staining for Iba-1 (green) and CD11b (red) in the hippocampus revealed that I/R-induced augmentation of Iba-1 or CD11b-positive cells was reduced in the I/R+si-ASK1 group at 7 days. **(C)** The graph shows the relative fluorescent intensities for Iba-1 and CD11b in the hippocampal CA1 region (*n* = 4). **(D)** The graph exhibits the relative fluorescent intensities for Iba-1 and CD11b in the hippocampal CA3 region (*n* = 4). Values are mean ± SD. Statistical comparisons between the groups were assessed with non-parametric Mann–Whitney test (^∗^*p* < 0.05, ^∗∗^*p* < 0.01, ^∗∗∗^*p* < 0.001 vs. I/R group). Statistical parameter (Supplementary Table 1). I/R, ischemia/reperfusion.

In hippocampus specimens stained with cresyl violet, cells in CA1 and CA3 were easily observed as violet-stained intact cell bodies. However, I/R decreased violet-stained cells in the CA1 and CA3 regions after 7 days. In contrast, ASK1 silencing by genetic manipulation inhibited the reduction of violet-stained cells caused by I/R injury (**Figure 2A**). Also, we evaluated the expression of Iba-1 and CD11b in the hippocampus, via immunofluorescent staining at 7 days after I/R (**Figure 2B**). In the control group, CA1 and CA3 regions of the hippocampus displayed small amounts of Iba-1-positive and CD11b-positive cells. In the I/R group, Iba-1-positive cells and CD11b-positive cells were largely founded in the CA1 and CA3 regions of the hippocampus. On the contrary, ASK1-silenced brain tissue showed decreased levels of Iba-1 and CD11b expression in CA1 and CA3, respectively. The graphs for relative fluorescent intensity showed enhanced intensities for Iba-1 and CD11b were efficiently diminished after silencing ASK1, compared with the I/R group (**Figures 2C,D**). These results indicated that ASK1 silencing leads to suppressed activation of microglia and macrophages in the hippocampus. Statistical parameters were shown in Supplementary Table 1.

### Silencing ASK1 Diminishes Pro-inflammatory and Increases Anti-inflammatory Mediators in Ischemic Brain Tissue

**FIGURE 3:**
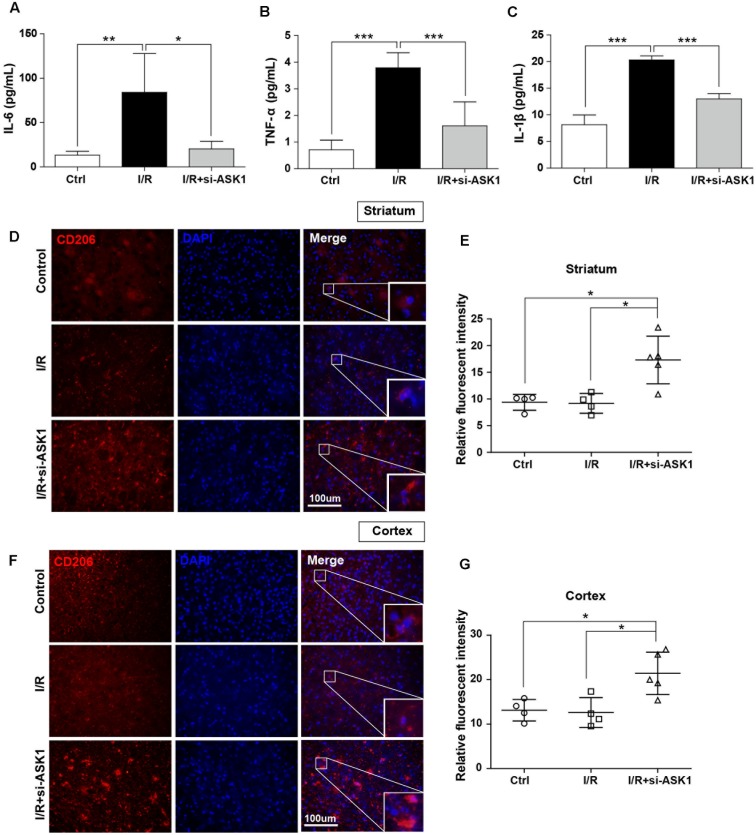
Decreased pro-inflammatory and increased anti-inflammatory mediators in the ischemic brain by silencing of ASK1 at 7 days after cerebral ischemia. The levels of **(A)** IL-6, **(B)** TNF-α, and **(C)** IL-1β in the hemisphere were assessed by ELISA at 7 days after I/R. I/R-induced upregulation of **(A)** IL-6, **(B)** TNF-α, and **(C)** IL-1β was decreased in the I/R+si-ASK1 group at 7 days (*n* = 8). Immunofluorescent staining for CD206 was performed at 7 days after I/R **(D,F)**. **(D)** The relative intensity graph indicates CD206-positive cells were increased in the striatum **(E)** and cortex **(G)**, compared with other groups (*n* = 4–5). Values are mean ± SD. Statistical comparisons between the groups were assessed with non-parametric Mann–Whitney test (^∗^*p* < 0.05, ^∗∗^*p* < 0.01, ^∗∗∗^*p* < 0.001 vs. I/R group). Statistical parameter (Supplementary Table 1). I/R, ischemia/reperfusion.

Several studies have demonstrated that microglia and macrophages can release pro-inflammatory mediators, such as IL-1β, IL-6, and TNF-α, under pathologic conditions. To investigate whether ASK1 silencing can reduce the release of pro-inflammatory cytokines, we assessed the expression there in the ischemic hemisphere at 7 days after I/R using ELISA (**Figure 3**). Our results showed that cerebral ischemia induced upregulation of IL-6, TNF-α, and IL-1β significantly, when compared with that of the control group. After silence of ASK1, increased levels of IL-6, TNF-α, and IL-1β were efficiently decreased in the ischemic hemisphere. Our ELISA data showed that ASK1 silencing reduces the expression of pro-inflammatory cytokines in the ischemic hemisphere (**Figures 3A–C**).

In addition, to examine whether ASK1 silencing can increase anti-inflammatory mediators, we performed immunofluorescent staining for CD206 (**Figure 3D**). Our results showed that CD206-positive cells in the control group were not significantly different between those in the I/R group; however, ASK1 silencing increased CD206-positive cells in the striatum and cortex (**Figures 3D–G**). To determine the effect of ASK1 on the cell fate, we performed TUNEL assay to detect DNA fragmentation at 7 days after I/R (Supplementary Figure 1C). The images and graphs showed that increased the number of TUNEL-positive cells in the striatum and cortex after I/R was efficiently reduced after ASK1 silencing. Therefore, our data showed that ASK1 silencing suppresses M1 phenotype and increases M2 phenotype in ischemic brain and reduces apoptotic cell death. Statistical parameters were shown in Supplementary Table 1.

### Inhibition of ASK1 Downregulates Pro-inflammatory Mediators, but Upregulates Anti-Inflammatory Mediators in Cultured BV2 Microglia Cell Line and Supernatants

**FIGURE 4:**
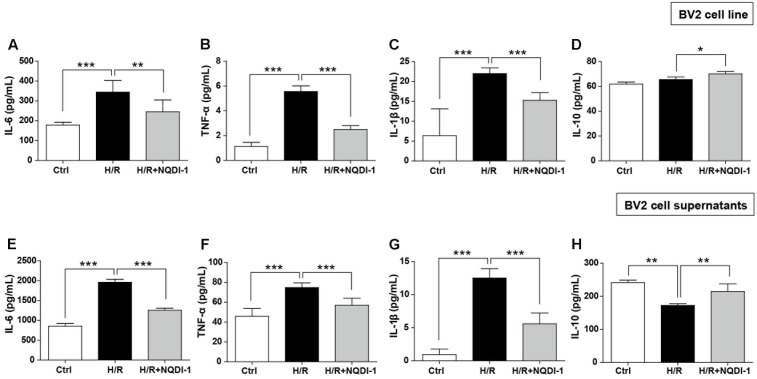
Downregulated pro-inflammatory cytokines and upregulated anti-inflammatory cytokines after inhibition of ASK1 in cultured BV2 microglia cell line and supernatants. The levels of pro-inflammatory mediators, such as **(A)** IL-6, **(B)** TNF-α, and **(C)** IL-1β and the anti-inflammatory mediator **(D)** IL-10 were determined by ELISA in BV2 cell line after H/R (4 h/24 h). Increased **(A)** IL-6 (7 repeats), **(B)** TNF-α (8 repeats), and **(C)** IL-1β (8 repeats) levels after H/R were reduced and **(D)** IL-10 (4 repeats) levels were increased by inhibition of ASK1 in BV2 cell line. The levels of **(E)** IL-6, **(F)** TNF-α, and **(G)** IL-1β and the anti-inflammatory mediator **(H)** IL-10 were determined by ELISA in BV2 microglial supernatants after H/R (4 h/24 h). Upregulated levels of **(E)** IL-6 (9 repeats), **(F)** TNF-α (8 repeats), and **(G)** IL-1β (8 repeats) after H/R were decreased and downregulated levels of **(H)** IL-10 (5 repeats) were upregulated by inhibition of ASK1 in BV2 microglial supernatants. Values are mean ± SD. Statistical comparisons between the groups were assessed with non-parametric Mann–Whitney test (^∗^*p* < 0.05, ^∗∗^*p* < 0.01, ^∗∗∗^*p* < 0.001 vs. H/R). Statistical parameter (Supplementary Table 2). H/R, hypoxia/reperfusion.

To investigate the effects of ASK1 on function of microglia, we performed single cell line cultures with BV2 cells and ELISA assay of cells (**Figures 4A–D**) and supernatants (**Figures 4E–H**). We used NQDI-1 drug *in vitro* to exclude effects of genetic manipulation unlike *in vivo* experiments. To identify the release of inflammatory mediators from BV2 microglia cell line, we also assayed cytokine levels in BV2 microglial supernatants. Our quantitative analysis showed that hypoxia/reperfusion (H/R) upregulated M1 phenotypes, reflected as release of IL-6, TNF-α, and IL-1β, both in BV2 microglia cell line (**Figures 4A–C**) and in BV2 microglial supernatants (**Figures 4E–G**). After treatment with NQDI-1 drug, an ASK1 inhibitor, the upregulated M1 mediators were efficiently reduced despite H/R injury. Although H/R could not alter IL-10 levels in BV2 microglia cell line (**Figure 4D**), compared with the control, NQDI-1 drug treatment upregulated secretion of IL-10 after H/R injury both in BV2 microglia cell line (**Figure 4D**) and in BV2 microglial supernatants (**Figure 4H**). Therefore, the results indicated that inhibition of ASK1 suppresses M1 phenotype and activates the alternative M2 phenotype in BV2 microglia cell line after H/R injury. Statistical parameters were shown in Supplementary Table 2.

### Suppression of ASK1 Decreases Pro-inflammatory Mediators, but Increases Anti-Inflammatory Mediators in Cultured RAW 264.7 Macrophage Cell Line and Supernatants

**FIGURE 5:**
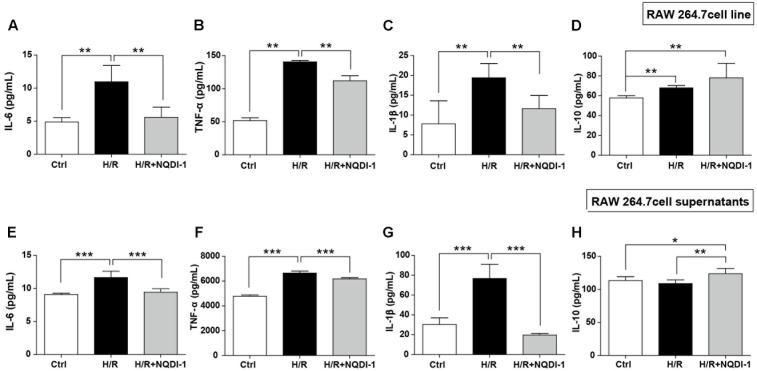
Downregulated pro-inflammatory cytokines and upregulated anti-inflammatory cytokines after inhibition of ASK1 in cultured RAW264.7 macrophage cell line and supernatants. The levels of pro-inflammatory mediators such as **(A)** IL-6, **(B)** TNF-α, and **(C)** IL-1β and the anti-inflammatory mediator **(D)** IL-10 were determined by ELISA in RAW 264.7 cell line after H/R (4 h/24 h). Increased **(A)** IL-6 (6 repeats), **(B)** TNF-α (5 repeats), and **(C)** IL-1β (6 repeats) levels after H/R were decreased and **(D)** IL-10 (5 repeats) levels were increased by inhibition of ASK1 in RAW264.7 cell line. The levels of **(E)** IL-6, **(F)** TNF-α, and **(G)** IL-1β, and the anti-inflammatory mediator **(H)** IL-10 were determined by ELISA in RAW264.7 supernatants after H/R (4 h/24 h). Augmented **(A)** IL-6 (6 repeats), **(B)** TNF-α (6 repeats), and **(C)** IL-1β (6 repeats) levels after H/R were diminished and **(D)** IL-10 (6 repeats) levels were increased by inhibition of ASK1 in RAW264.7 supernatants. Values are mean ± SD. Statistical comparisons between the groups were assessed with non-parametric Mann–Whitney test (^∗^*p* < 0.05, ^∗∗^*p* < 0.01, ^∗∗∗^*p* < 0.001 vs. H/R). Statistical parameter (Supplementary Table 2). H/R, hypoxia/reperfusion.

To examine the effects of ASK1 on macrophage function, we performed single cell line cultures with RAW 264.7 cells and ELISA of cells and supernatants (**Figure 5**). Our results suggested that H/R injury triggered expression of M1-related mediators, such as IL-6, TNF-α, and IL-1β. However, suppression of ASK1 significantly decreased M1 phenotypes in RAW 264.7 cell line (**Figures 5A–C**) and in supernatants (**Figures 5E–G**). In addition, IL-10 levels were also increased after inhibition of ASK1 in spite of H/R injury, as compared with control and H/R injured macrophages, both in RAW 264.7 cell line (**Figure 5D**) and in supernatants (**Figure 5H**). Therefore, our data showed that inhibition of ASK1 reduces M1 phenotype and increases M2 phenotype in RAW 264.7 macrophage cell line. Statistical parameters were shown in Supplementary Table 2.

### Inhibition of ASK1 Induces Transcription of M2 Polarization in BV2 Microglia and RAW 264.7 Macrophage Cell Lines

**FIGURE 6:**
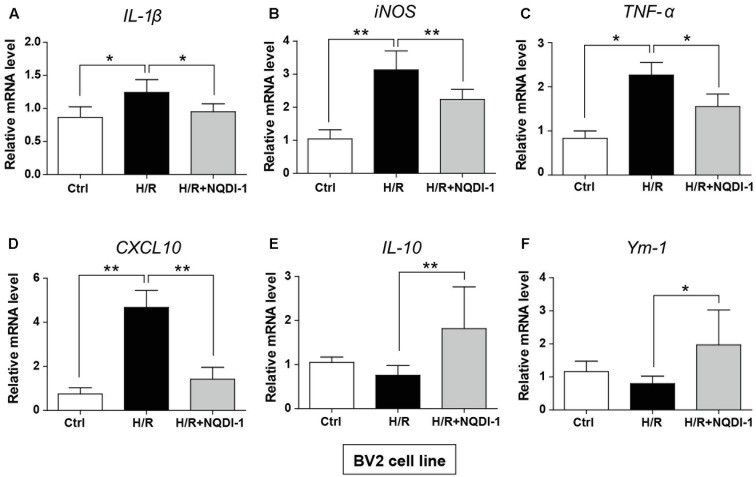
Changes in M1 and M2 specific genes after inhibition of ASK1 in BV2 microglia cell line. The mRNA levels of M1 specific markers, such as **(A)** IL-1β (5 repeats), **(B)** iNOS (6 repeats), **(C)** TNF-α (5 repeats), and **(D)** CXCL10 (6 repeats), and M2 specific markers, such as **(E)** IL-10 (5 repeats), and **(F)** Ym-1 (4-5 repeats), were measured by real-time PCR in BV2 cell line after H/R (4 h/24 h). Increased mRNA levels of **(A)** IL-1β, **(B)** iNOS, **(C)** TNF-α, and **(D)** CXCL10 were downregulated, while **(E)** IL-10 and **(F)** Ym-1 levels were increased by inhibition of ASK1 in BV2 cell line. Values are mean ± SD. Statistical comparisons between the groups were assessed with non-parametric Mann–Whitney test (^∗^*p* < 0.05, ^∗∗^*p* < 0.01, ^∗∗∗^*p* < 0.001 vs. H/R group). Statistical parameter (Supplementary Table 3). H/R, hypoxia/reperfusion.

**FIGURE 7:**
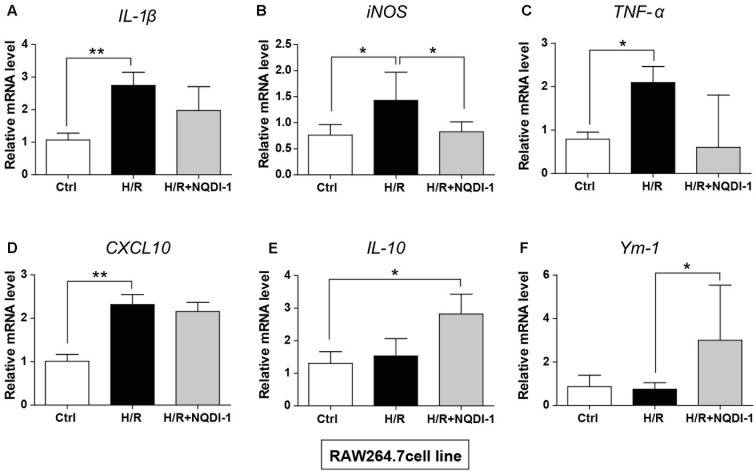
Changes in M1 and M2 specific genes after inhibition of ASK1 in RAW264.7 macrophage cell line. The mRNA levels of M1 specific markers, such as **(A)** IL-1β (6 repeats), **(B)** iNOS (4–5 repeats), **(C)** TNF-α (4 repeats), and **(D)** CXCL10 (6 repeats), and M2 specific markers, such as **(E)** IL-10 (4 repeats) and **(F)** Ym-1 (5–6 repeats), were measured by real-time PCR in RAW264.7 cell line after H/R (4 h/24 h). The mRNA levels of **(A)** IL-1β, **(B)** iNOS, **(C)** TNF-α, and **(D)** CXCL10 were upregulated after H/R. The levels of iNOS were reduced, while **(E)** IL-10 and **(F)** Ym-1 levels were upregulated by inhibition of ASK1 in RAW264.7 cell line. Values are mean ± SD. Statistical comparisons between the groups were assessed with non-parametric Mann–Whitney test (^∗^*p* < 0.05, ^∗∗^*p* < 0.01, ^∗∗∗^*p* < 0.001 vs. H/R). Statistical parameter (Supplementary Table 3). H/R, hypoxia/reperfusion.

Genetic expression of M1 and M2 markers was quantified by real-time PCR in BV2 microglia (**Figure 6**) and in RAW 264.7 macrophage cell lines (**Figure 7**). Similar to cytokine levels evaluated by ELISA, the mRNA levels of M1 polarization-related factors such as IL-1β, TNF-α, inducible nitric oxide (iNOS), and CXCL10 were all significantly increased in BV2 microglia cell line after H/R injury (**Figures 6A–D**), and those of M2 polarization-related factors such as IL-10 and Ym-1 showed no significant difference (**Figures 6E,F**). After treatment with NQDI-1 drug, mRNA levels of IL-1β, iNOS, TNF-α, and CXCL10 were all efficiently downregulated despite H/R injury (**Figures 6A–D**). The expression of M2-related factors including IL-10 and Ym-1 were remarkably higher in ASK1-inhibited BV2 microglia cell line (**Figures 6E,F**). Selected expression of M1 and M2 phenotypes was quantified by real-time PCR in RAW 264.7 macrophage cell line (**Figure 7**). As in the case of cytokine expression using ELISA, H/R induced mRNA levels of IL-1β, TNF-α, iNOS, and CXCL10 in macrophage cell line (**Figures 7A–D**). Treatment with NQDI-1 drug efficiently decreased mRNA levels of iNOS. Further, ASK1 inhibition induced anti-inflammatory IL-10 and Ym-1 mRNA levels, whereas H/R injury triggered M1-polarization (**Figures 7E,F**). Therefore, our data suggested that inhibition of ASK1 promotes BV2 microglia and RAW 264.7 macrophage cell lines toward M2 polarization after H/R injury. Statistical parameters were shown in Supplementary Table 3.

### Inhibition of ASK1 Increases M2 Phenotype of Microglia and Macrophages

**FIGURE 8:**
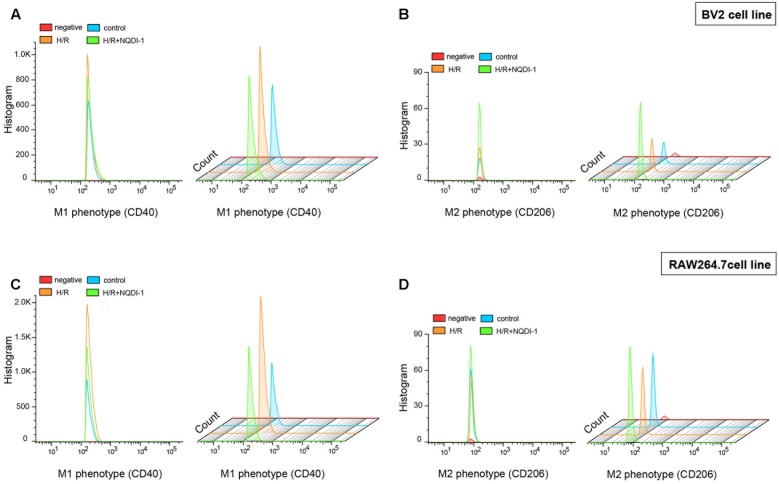
Alteration of M1 and M2 surface markers after inhibition of ASK1 in BV2 microglia and RAW264.7 macrophages. Flow cytometry was performed for discrimination of **(A)** M1 (CD40) or **(B)** M2 (CD206) positive cell populations of BV2 cell line after H/R (4 h/24 h). The histogram of flow cytometry shows **(C)** M1 (CD40) and **(D)** M2 (CD206) positive cells in RAW 264.7 cell line after H/R (4 h/24 h). H/R, hypoxia/reperfusion.

To confirm the expression of cell surface markers, we performed flow cytometry using BV2 microglia and RAW 264.7 macrophage cell lines. In this study, CD40 and CD206 were used as M1 and M2 markers, respectively (**Figure 8**). In BV2 cells, histograms showed that the expression of the M1 marker CD40 was highly present after H/R injury. However, NQDI-1 drug treatment reduced the expression of CD40-positive cells after H/R (**Figure 8A**). In the analysis of M2 phenotype, M2 marker (CD206-positive cells) was significantly increased on NQDI-1 drug treatment (**Figure 8B**). Moreover, in RAW 264.7 cells, histograms revealed higher counts of CD40-positive cells after H/R challenge, whereas inhibition of ASK1 decreased CD40-positive cell counts despite H/R injury (**Figure 8C**). In contrast, after suppression of ASK1 with NQDI-1 drug, the expression of CD206-positive cells was higher in the H/R stimulated RAW 264.7 cell line (**Figure 8D**). Statistical parameters were shown in Supplementary Table 3.

### Microglia and Macrophage Hyper-Activation Is Reduced after Inhibition of ASK1

**FIGURE 9:**
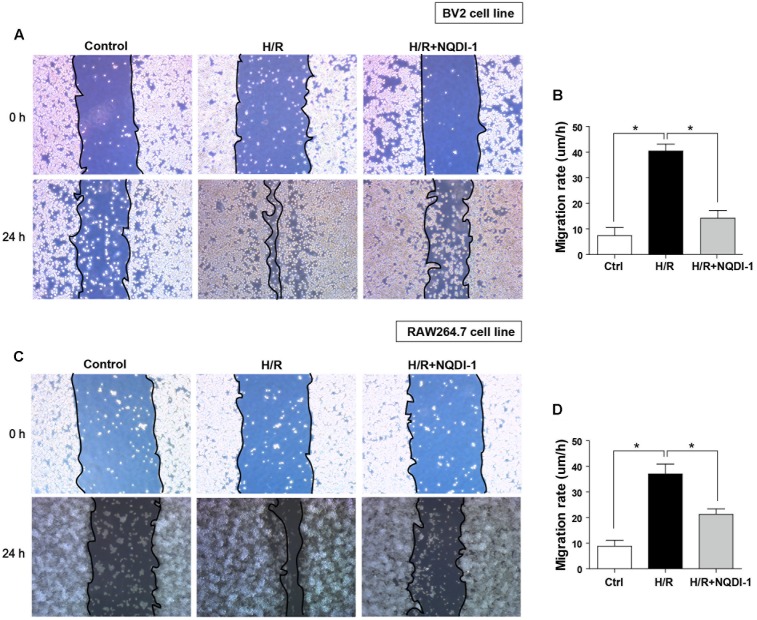
Function of BV2 microglia and RAW264.7 macrophages after inhibition of ASK1. **(A,B)** Gap closure assay was performed for BV2 and RAW264.7 cells **(A)** Images at 0 h after hypoxia and 24 h after hypoxia/reperfusion in BV2 cell line. **(B)** The graph represents that H/R-induced increases in migration rate were decreased by inhibition of ASK1 in BV2 cell line (4 repeats). **(C)** Images at 0 h after hypoxia and 24 h after hypoxia/reperfusion in RAW264.7 cell line. **(D)** The graph represents that H/R-induced increases in migration rate were reduced by inhibition of ASK1 in RAW264.7 cell line (4 repeats). Values are mean ± SD. Statistical comparisons between the groups were assessed with non-parametric Mann–Whitney test (^∗^*p* < 0.05, ^∗∗^*p* < 0.01, ^∗∗∗^*p* < 0.001 vs. H/R). Statistical parameter (Supplementary Table 3). H/R, hypoxia/reperfusion.

To analyze cell migration, we performed gap closure assay in cultured BV2 microglia and RAW 264.7 macrophage cell lines (**Figure 9**). Before H/R injury, we made a gap with a pipette tip, and cell debris was washed away with DPBS. We observed BV2 microglia cell line under a microscope immediately after H/R injury and at 24 h after H/R injury (**Figure 9A**). H/R injury increased migration rates of BV2 microglia cell line. However, inhibition of ASK1 reduced migration rates (**Figure 9B**). The same methods were used in RAW 264.7 macrophage cell culture. We observed RAW 264.7 cell line under a microscope right after H/R injury and at 24 h after H/R injury (**Figure 9C**). A high rate of migration was exhibited in H/R-injured macrophages; however, NQDI-1 drug treatment retarded the migration rate. Therefore, ASK1 is associated with microglia and macrophage migration capacity and ASK1 inhibition delayed migration rate. Statistical parameters were shown in Supplementary Table 3.

## Discussion

In this study, we performed *in vivo* and *in vitro* ischemic stroke model to examine whether ASK1 modulates microglia/macrophage polarization and function. We proved that ASK1 controls polarization and function of microglia/macrophage after ischemic/hypoxic injury. In previous reports, ASK1 is one of the early responders to different types of stress in the intracellular system, including oxidative stress, calcium overload, and immune response (Hayakawa et al., 2006; Takeda et al., 2008). ASK1 governs activation of mitogen-activated protein kinase (MAP kinase), which is essential for cellular function (Shiizaki et al., 2013), and several studies support that ASK1 is closely related to cerebral ischemia (Kim et al., 2011; Cheon et al., 2016b). Under inflammatory conditions, ASK1 signaling is necessary for TLRs, which recognize LPS (Soga et al., 2012). ASK1 deficiency represents LPS resistance, and genetic deletion of ASK1 involves inhibition of pro-inflammatory mediator production (Matsukawa et al., 2004; Sumbayev, 2008; Takeda et al., 2008; Mnich et al., 2010). The ASK1/p38 signaling pathway via TLRs is critical for chemokine production and promotes inflammation and neurotoxicity after multiple sclerosis (Guo et al., 2010). In line with studies that have demonstrated that ASK1 inhibition attenuates pro-inflammatory cytokines in microglia, ASK1 was found to be associated with TNF-α and iNOS production in primary microglial cell culture, and M1 macrophage suppression by a Class A scavenger receptor resulted in reductions of IL-6, IL-1β, and TNF-α, decreasing ASK1/p38/NF-κB pathway signaling, under pathologic conditions (Hu et al., 2011; Katome et al., 2013; Song and Lee, 2015). Previous reports have shown that inhibition of ASK1 reduces M1 phenotype after injury and ASK1 also played a role in our results. The main findings of this study are (1) in the ipsilateral hemisphere, ASK1 silencing resulted in a reduction of ischemic-induced activation of microglia/infiltrated macrophages and increased M2 phenotype in the late phase of cerebral ischemia and (2) ASK1 inhibition polarized BV2 microglia and RAW 264.7 macrophage cell lines toward M2 phenotype after hypoxia/reperfusion injury. Our *in vivo* study revealed that cerebral ischemia promotes activation of microglia and infiltration of macrophages in brain regions. In the ipsilateral hemisphere, upregulation of pro-inflammatory cytokines, such as IL-6, TNF-α, and IL-1β, was observed. However, ASK1 silenced by siRNA downregulated activation of microglia/macrophage and pro-inflammatory cytokine levels and upregulated the anti-inflammatory mediators. Our *in vitro* study for M1/M2 polarization of microglia/macrophages revealed that hypoxic injury stimulates M1-related factors, while ASK1 inhibition by NQDI-1 drug suppressed M1-related factors and promoted M2-related factors in microglia and macrophage cell lines. In addition, inhibition of ASK1 retarded the migration rate of both microglia and macrophages in gap closure assay.

Inflammation has emerged as the key factor in progression of ischemic stroke, and the inflammatory mediators play an important role in enlargement of brain damage and neurological dysfunction (Matsukawa et al., 2004; Quan and Banks, 2007; Quan, 2008; Banks and Erickson, 2010; Fu et al., 2015; Anrather and Iadecola, 2016). At the onset of ischemic stroke, stagnant blood flow initiates an aberrant immune response that promotes infiltration of immune cells such as leukocytes and macrophages, via a disrupted BBB (Matsukawa et al., 2004; Quan and Banks, 2007; Quan, 2008; Banks and Erickson, 2010; Fu et al., 2015; Anrather and Iadecola, 2016). Under the altered environment, microglia are activated. Meanwhile, the morphology and activation of peripherally infiltrated macrophages in the brain changes depending on the extracellular stimuli (Durafourt et al., 2012; Hu et al., 2015; Orihuela et al., 2016), after which activated microglia migrate quickly toward lesion sites, and lead to cell accumulation by releasing inflammation-associated mediators (Danton and Dietrich, 2003). Based on the microenvironment, microglia/macrophage can switch between phenotypes M1 (classically activated) and M2 (alternative activated). Under M1 conditions stimulated by LPS or IFN-γ, M1 microglia/macrophage express pro-inflammatory mediators of IL-1β, IL-6, TNF-α, iNOS, and CXCL10 and the cell surface markers CD40, CD80, and CD86. Also, cytotoxic effects of the M1 phenotype can exacerbate tissue damage (Durafourt et al., 2012; Hu et al., 2015; Kapellos and Iqbal, 2016; Orihuela et al., 2016; Plastira et al., 2016). On the contrary, M2 microglia/macrophages exert anti-inflammatory responses by upregulating IL-10, arginase-1, Ym-1 (heparin-binding lectin), and mannose receptor CD206, which modulate tissue repair, regeneration, and remodeling (Durafourt et al., 2012; Barakat and Redzic, 2016; Orihuela et al., 2016). During the early stage after ischemic insult, expression of the pro-inflammatory cytokine TNF-α is increased in neurons. However, during the late phase of ischemic insult, it is augmented in microglia/macrophage and other immune cells, with microglia/macrophage being major sources of pro-inflammatory cytokine in ischemic lesions after ischemic stroke (Gregersen et al., 2000; Clausen et al., 2008). Late inflammation in the brain after ischemic stroke is represented by persistent immune cell infiltration and cerebral infarctions (Mena et al., 2004; Brea et al., 2009; Vogelgesang et al., 2014; Walter et al., 2015; Anrather and Iadecola, 2016). Several studies have reported that microglia/macrophage shift their phenotype from M2 to M1 after cerebral ischemia and M1 microglia/macrophage dominate the ischemic lesion in the brain, which exacerbate brain injury (Frieler et al., 2011; Hu et al., 2012). M1 polarized microglia/macrophage during chronic inflammation augment neuronal damage and block reestablishment of neuronal network, thereby retarding brain recovery (Hu et al., 2012). However, M2 polarized microglia/macrophage show improved phagocytic activity for clearance of necrotic debris and ameliorate production of inflammatory mediators (Hu et al., 2012). M2 phenotype modifies the extracellular matrix and promotes axonal regeneration and angiogenesis (Kigerl et al., 2009). In addition, after ischemic/hypoxic insult, M2 microglia/macrophage mediates clearance of ischemic tissue and blockade of brain injury, leading to neuronal survival (Hu et al., 2012). Therefore, anti-inflammatory M2 may have beneficial effects on ameliorating the development of brain damage. In the current study, cerebral ischemia augmented the recruitment of macrophage and microglia activation and migration, and led the brain toward M1 environment. However, ASK1 silencing by si-RNA/ASK1 inhibition by NQDI-1 drug diminished M1 phenotype-specific markers (secretion of pro-inflammatory mediators and downregulation of pro-inflammatory mediator genes) of macrophage/microglia and upregulated M2 specific markers (expression of anti-inflammatory mannose receptor and upregulation of anti-inflammatory mediator genes) in the later stages of the recovery period after ischemic/hypoxic injuries. To our knowledge, this is the first investigations of ASK1 in regards to M1/M2 polarization of microglia and macrophages in the late stage of ischemic stroke.

However, our limitations are as follows: first, it is not clarified which ASK1-downstream or -related signaling pathway affects M1/M2 phenotype. Although several molecules (JNK, p38, and Akt) are known to be involved in polarization (Zhang and Zhang, 2015; Vergadi et al., 2017; Zhong et al., 2017), we only proceeded to examine changes of M1/M2 phenotype by silencing/inhibiting ASK1 levels. Second, we focused on “classical” M1/M2 polarization in this study. However, Martinez et al. (2008) and Mantovani et al. (2004) review the subpopulation of polarization, such as M1, M2a, M2b, and M2c. Ransohoff (2016) raises some concerns on existence of M1/M2 polarization (Mantovani et al., 2004; Martinez et al., 2008). Third, the previous studies proved that ASK1 inhibitor NQDI-1 shows a possible therapeutic application as a protective drug in ischemic stroke (Song et al., 2015; Cheon et al., 2016a). However, it is not fully demonstrated its safety and application. If this limitation is resolved, it will be one of the candidates for use in ischemic stroke patients.

## Conclusion

We showed that ASK1 mediates M1/M2 polarization and the functions of microglia and macrophages. Modulation of the late inflammatory environment by M1/M2 regulation of microglia and macrophage via ASK1 silencing after cerebral ischemia may be an attractive strategy for recovery from stroke.

## Author Contributions

B-NK designed this study, supervised the project, interpreted all data, and wrote the manuscript. SC participated in the collection of data, interpretation of data, and writing of the first draft of the manuscript. EJK, JK, EHK, and BK participated in the data collection and interpretation.

## Conflict of Interest Statement

The authors declare that the research was conducted in the absence of any commercial or financial relationships that could be construed as a potential conflict of interest.
