# Supplementary material for: Regulation of Microglia and Macrophage Polarization via Apoptosis Signal-Regulating Kinase 1 Silencing after Ischemic/Hypoxic Injury
Source: Front Mol Neurosci. 2017 Aug 14;10:261. doi: 10.3389/fnmol.2017.00261 (PMC5557792; doi:10.3389/fnmol.2017.00261)
Supplement: Supplementary file 1 [file Data_Sheet_1.pdf]

[Supplementary data]

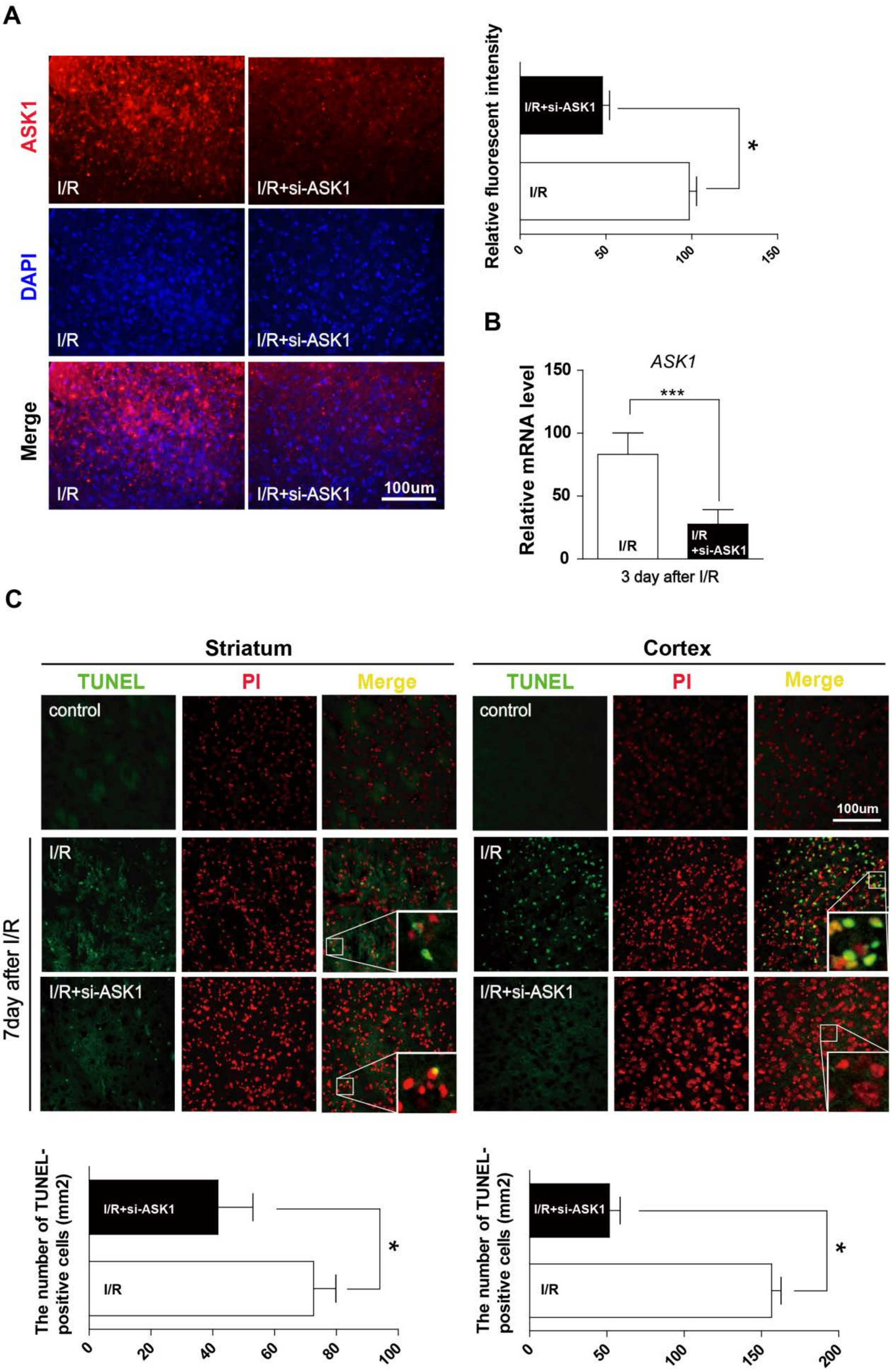

Supplementary Figure 1. (A) ASK1 expressions (red) were efficiently decreased in ipsilateral hemisphere after ASK1 silencing at 7 days after I/R, compared with the I/R group (n = 4). (B) ASK mRNA levels were significantly reduced in ipsilateral hemisphere after ASK1 silencing by genetic manipulation at 3 days after I/R, compared with the I/R group (n = 7). (C) DNA fragmentation in the striatum and cortex was detected by TUNEL assay. The graphs show that TUNEL-positive cells were decreased in the striatum and cortex after ASK1 silencing, compared with the I/R group (n = 4). Values are means ± SD. Statistical comparisons between the groups were assessed with non-parametric Mann-whitney test (* $p < 0.05$, ** $p < 0.01$, *** $p < 0.001$ vs I/R group). Statistical parameter (Supplementary Table 3). I/R, ischemia/reperfusion.

Table 1. Statistical parameter in Figure 1, 2, and 3.

Immunohistochemistry

Iba-1 (striatum)	control	I/R	I/R+si-ASK1
Mean	2.524	14.51	3.469
Std. Deviation	0.5919	3.894	1.339
**Iba-1 (cortex)**	**control**	**I/R**	**I/R+si-ASK1**
Mean	1.929	12.5	2.731
Std. Deviation	1.601	3.104	0.5195
**CD11b (striatum)**	**control**	**I/R**	**I/R+si-ASK1**
Mean	0.8098	8.661	1.236
Std. Deviation	0.2241	1.513	0.4365
**CD11b (cortex)**	**control**	**I/R**	**I/R+si-ASK1**
Mean	0.778	8.983	1.428
Std. Deviation	0.5088	2.179	0.4768
**Iba-1 (CA1)**	**control**	**I/R**	**I/R+si-ASK1**
Mean	3.063	13.34	4.783
Std. Deviation	0.3315	1.631	2.36
**Iba-1 (CA3)**	**control**	**I/R**	**I/R+si-ASK1**
Mean	2.778	21.97	6.179
Std. Deviation	0.9622	6.926	1.311
**CD11b (CA1)**	**control**	**I/R**	**I/R+si-ASK1**
Mean	2.295	8.614	3.025
Std. Deviation	1.138	0.7854	2.574
**CD11b (CA3)**	**control**	**I/R**	**I/R+si-ASK1**
Mean	1.63	9.314	1.713
Std. Deviation	0.5218	2.318	0.6213

ELISA (ipsilateral hemisphere)

IL-6	control	I/R	I/R+si-ASK1
Mean	13.36	84.07	20.31
Std. Deviation	4.373	43.79	8.612
**TNF-α**	**control**	**I/R**	**I/R+si-ASK1**
Mean	0.7096	3.79	1.61
Std. Deviation	0.3641	0.5672	0.9012
**IL-1β**	**control**	**I/R**	**I/R+si-ASK1**
Mean	8.146	20.29	12.99
Std. Deviation	1.818	0.79	1.002

Immunohistochemistry

CD206 (striatum)	control	I/R	I/R+si-ASK1
Mean	9.377	9.176	17.31
Std. Deviation	1.483	1.845	4.47
**CD206 (cortex)**	**control**	**I/R**	**I/R+si-ASK1**
Mean	13.13	12.6	21.43
Std. Deviation	2.414	3.363	4.755

Table 2. Statistical parameter in Figure 4 and 5.

ELISA (BV2 cell line)

IL-6	control	I/R	I/R+si-ASK1
Mean	178.3	344.2	245.4
Std. Deviation	13.95	59.13	59.27
TNF-α	control	I/R	I/R+si-ASK1
Mean	1.124	5.565	2.503
Std. Deviation	0.3419	0.4499	0.3077
IL-1β	control	I/R	I/R+si-ASK1
Mean	6.372	22.01	15.29
Std. Deviation	6.739	1.426	1.912
IL-10	control	I/R	I/R+si-ASK1
Mean	61.93	65.56	70.07
Std. Deviation	1.583	2.076	1.871

ELISA (RAW264.7 cell line)

IL-6	control	I/R	I/R+si-ASK1
Mean	4.884	10.97	5.591
Std. Deviation	0.6582	2.467	1.528
TNF-α	control	I/R	I/R+si-ASK1
Mean	51.63	140.6	112
Std. Deviation	4.341	2.177	7.673
IL-1β	control	I/R	I/R+si-ASK1
Mean	7.777	19.4	11.62
Std. Deviation	5.814	3.592	3.305
IL-10	control	I/R	I/R+si-ASK1
Mean	57.78	67.96	78.07
Std. Deviation	2.226	2.426	14.65

ELISA (BV2 cell supernatants)

IL-6	control	I/R	I/R+si-ASK1
Mean	853	1959	1255
Std. Deviation	65.69	75.97	50.76
TNF-α	control	I/R	I/R+si-ASK1
Mean	45.85	74.78	57
Std. Deviation	7.755	4.661	7.019
IL-1β	control	I/R	I/R+si-ASK1
Mean	0.9468	12.5	5.582
Std. Deviation	0.8164	1.415	1.635
IL-10	control	I/R	I/R+si-ASK1
Mean	241.5	172.3	214.7
Std. Deviation	7.022	5.66	22.93

ELISA (RAW264.7 cell supernatants)

IL-6	control	I/R	I/R+si-ASK1
Mean	9.065	11.64	9.434
Std. Deviation	0.2011	0.9641	0.5123
TNF-α	control	I/R	I/R+si-ASK1
Mean	4774	6651	6180
Std. Deviation	110.9	158	93.75
IL-1β	control	I/R	I/R+si-ASK1
Mean	30.38	76.72	19.64
Std. Deviation	6.633	14.3	1.736
IL-10	control	I/R	I/R+si-ASK1
Mean	113.4	108.8	123.7
Std. Deviation	5.858	5.508	7.919

Table 3. Statistical parameter in Figure 6, 7, 8 and Supplementary Figure 1.

Real-time (BV2 cell line)

IL-1β	control	I/R	I/R+si-ASK1
Mean	0.8649	1.24	0.9491
Std. Deviation	0.1597	0.1947	0.1211
iNOS	control	I/R	I/R+si-ASK1
Mean	1.04	3.131	2.237
Std. Deviation	0.2771	0.5732	0.3029
TNF-α	control	I/R	I/R+si-ASK1
Mean	0.8347	2.267	1.554
Std. Deviation	0.1677	0.2814	0.2825
CXCL10	control	I/R	I/R+si-ASK1
Mean	0.7453	4.676	1.417
Std. Deviation	0.2891	0.7727	0.5403
IL-10	control	I/R	I/R+si-ASK1
Mean	1.048	0.7553	1.817
Std. Deviation	0.1236	0.224	0.9471
Ym-1	control	I/R	I/R+si-ASK1
Mean	1.159	0.7947	1.97
Std. Deviation	0.317	0.2259	1.056

Real-time (RAW264.7 cell line)

IL-1β	control	I/R	I/R+si-ASK1
Mean	1.067	2.747	1.973
Std. Deviation	0.2124	0.4003	0.7327
iNOS	control	I/R	I/R+si-ASK1
Mean	0.7636	1.431	0.8261
Std. Deviation	0.2042	0.5407	0.1908
TNF-α	control	I/R	I/R+si-ASK1
Mean	0.7923	2.096	0.6033
Std. Deviation	0.1623	0.3692	1.203
CXCL10	control	I/R	I/R+si-ASK1
Mean	1.01	2.315	2.153
Std. Deviation	0.1594	0.2321	0.2142
IL-10	control	I/R	I/R+si-ASK1
Mean	1.309	1.53	2.822
Std. Deviation	0.3549	0.535	0.6097
Ym-1	control	I/R	I/R+si-ASK1
Mean	0.8619	0.7446	3.001
Std. Deviation	0.5326	0.3041	2.541

Migration rates

BV2 cell line	control	I/R	I/R+si-ASK1
Mean	7.743	40.87	14.04
Std. Deviation	2.679	2.352	2.983
RAW264.7 cell line	control	I/R	I/R+si-ASK1
Mean	8.585	37.66	21.54
Std. Deviation	1.952	3.436	1.821

TUNEL assay

cortex	I/R	I/R+si-ASK1
Mean	51.67	14.04
Std. Deviation	6.939	2.983
striatum	I/R	I/R+si-ASK1
Mean	72.65	41.67
Std. Deviation	7.236	11.39

Immunohistochemistry

ASK1	I/R	I/R+si-ASK1
Mean	98.51	47.97
Std. Deviation	4.25	4.184

Real-time

ASK1	I/R	I/R+si-ASK1
Mean	83.24	27.68
Std. Deviation	17.01	11.56
